# Poly[μ-aqua-bis­(μ_5_-2,4-di­chloro­benzoato)dipotassium]

**DOI:** 10.1107/S1600536813033503

**Published:** 2013-12-18

**Authors:** Graham Smith

**Affiliations:** a Science and Engineering Faculty, Queensland University of Technology, GPO Box 2434, Brisbane, Queensland 4001, Australia

## Abstract

In the title compound, [K_2_(C_7_H_3_Cl_2_O_2_)_2_(H_2_O)]_*n*_, the potassium salt of 2,4-di­chloro­benzoic acid, the repeating unit in the polymeric structure consists of two identical irregular KO_6_Cl units related by twofold rotational symmetry, linked by a bridging water mol­ecule lying on the twofold axis. The coordination polyhedron about the K^+^ ion comprises a carboxyl­ate O atom and a Cl-atom donor from a bidentate chelate ligand inter­action, four O-atom donors from a doubly bridging bidentate carboxyl­ate *O*,*O*′-chelate inter­action and the water mol­ecule. A two-dimensional polymeric structure lying parallel to (100) is generated through a series of conjoined cyclic bridges between K^+^ ions and is stabilized by water–carboxyl­ate O—H⋯O hydrogen-bonding inter­actions.

## Related literature   

For the structures of potassium salts with coordinating carbon-bound Cl ligands, see: Gowda *et al.* (2007 ▶); Molčanov *et al.* (2011 ▶). For an analogous complex with a Cs—Cl bond in a bidentate chelate mode, see: Smith (2013 ▶). For the structure of ammonium 2,4-di­chloro­benzoate, see: Smith (2014 ▶).
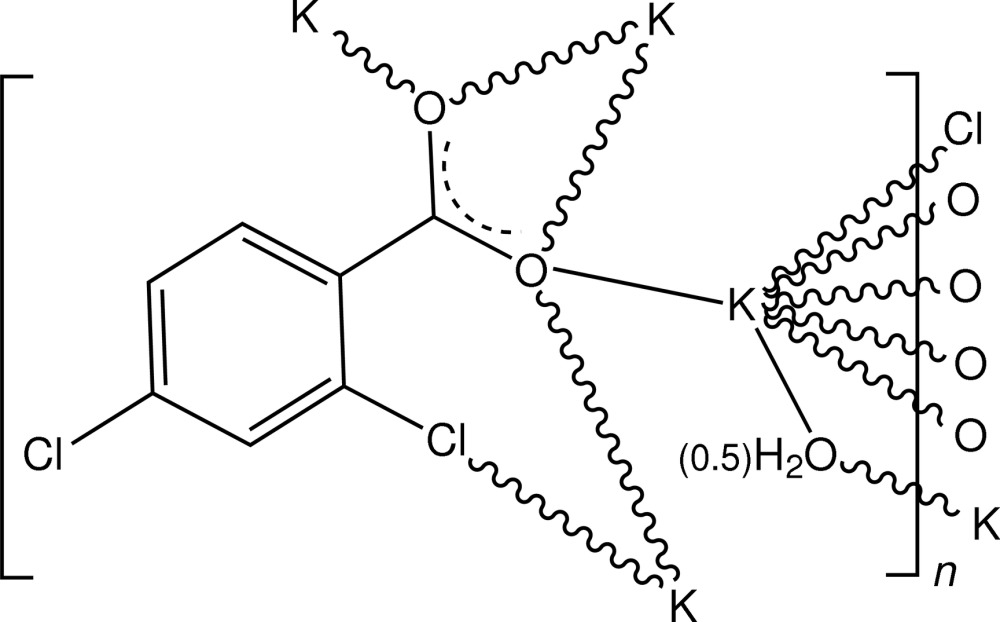


## Experimental   

### 

#### Crystal data  

[K_2_(C_7_H_3_Cl_2_O_2_)_2_(H_2_O)]
*M* *_r_* = 476.20
Monoclinic, 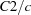

*a* = 31.520 (2) Å
*b* = 4.3407 (3) Å
*c* = 12.7849 (9) Å
β = 94.427 (6)°
*V* = 1744.0 (2) Å^3^
*Z* = 4
Mo *K*α radiation
μ = 1.18 mm^−1^
*T* = 200 K
0.35 × 0.35 × 0.04 mm

#### Data collection  

Oxford Diffraction Gemini-S CCD diffractometer
Absorption correction: multi-scan (*CrysAlis PRO*; Agilent, 2012 ▶) *T*_min_ = 0.706, *T*_max_ = 0.980
9909 measured reflections
1714 independent reflections
1534 reflections with *I* > 2σ(*I*)
*R*_int_ = 0.084

#### Refinement  

*R*[*F* ^2^ > 2σ(*F* ^2^)] = 0.031
*wR*(*F* ^2^) = 0.081
*S* = 1.09
1714 reflections
114 parameters
H-atom parameters constrained
Δρ_max_ = 0.37 e Å^−3^
Δρ_min_ = −0.23 e Å^−3^

### 

Data collection: *CrysAlis PRO* (Agilent, 2012 ▶); cell refinement: *CrysAlis PRO*; data reduction: *CrysAlis PRO*; program(s) used to solve structure: *SHELXS97* (Sheldrick, 2008 ▶); program(s) used to refine structure: *SHELXL97* (Sheldrick, 2008 ▶) within *WinGX* (Farrugia, 2012 ▶); molecular graphics: *PLATON* (Spek, 2009 ▶); software used to prepare material for publication: *PLATON*.

## Supplementary Material

Crystal structure: contains datablock(s) global, I. DOI: 10.1107/S1600536813033503/wm2791sup1.cif

Structure factors: contains datablock(s) I. DOI: 10.1107/S1600536813033503/wm2791Isup2.hkl

Supporting information file. DOI: 10.1107/S1600536813033503/wm2791Isup3.cml

Additional supporting information:  crystallographic information; 3D view; checkCIF report

## Figures and Tables

**Table 1:** Selected bond lengths (Å)

K1—O1*W*	2.7597 (12)
K1—O12	2.7443 (15)
K1—Cl2^i^	3.2670 (7)
K1—O12^i^	2.7699 (15)
K1—O11^ii^	3.0826 (14)
K1—O12^ii^	2.8168 (14)
K1—O11^iii^	2.7815 (15)

Symmetry codes: (i) 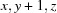; (ii) 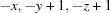; (iii) 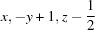.

**Table 2:** Hydrogen-bond geometry (Å, °)

*D*—H⋯*A*	*D*—H	H⋯*A*	*D*⋯*A*	*D*—H⋯*A*
O1*W*—H11*W*⋯O11^iv^	0.81	1.92	2.7271 (19)	169

Symmetry code: (iv) 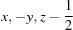.

**Fig. 1.**
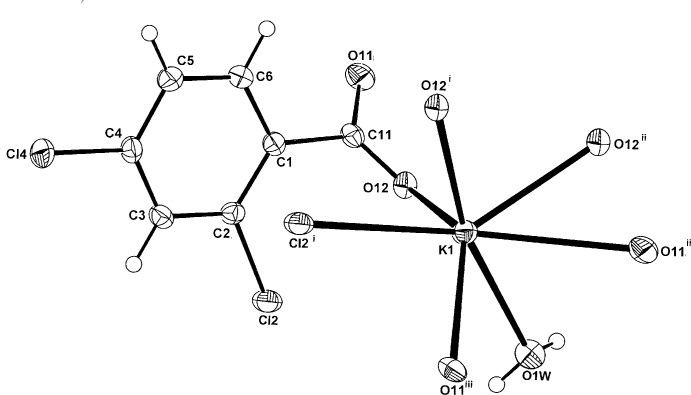
The configuration and atom-numbering scheme for the coordination polyhedron of the title complex, with non-H atoms drawn as 40% probability displacement ellipsoids. The bridging water molecule (O1W) lies on a twofold rotation axis. For symmetry codes, see: Table 1.

**Fig. 2.**
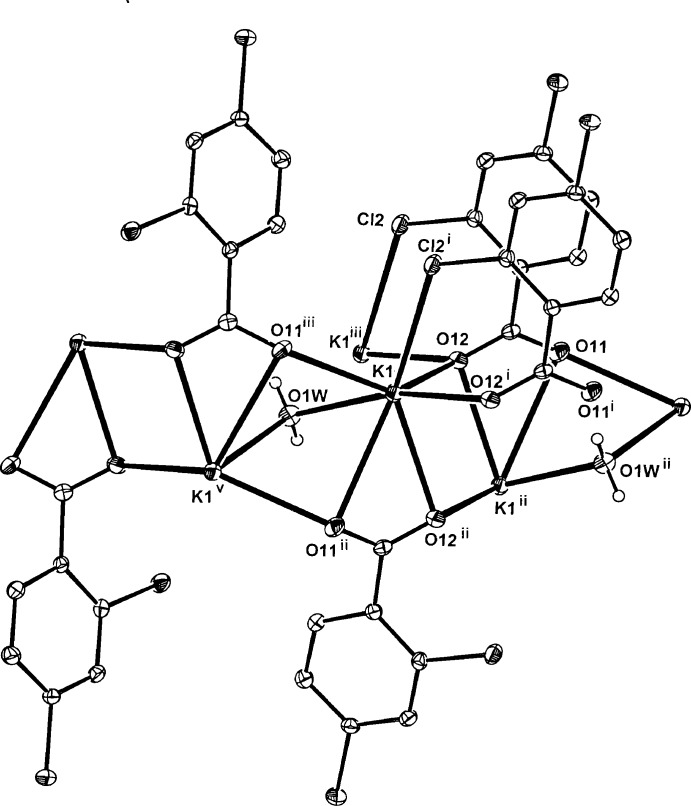
A partial expansion of the KO6Cl coordination sphere in the polymeric structure. Probability code as in Fig. 1. For symmetry code (v): -x, y, -z + 1/2. For other symmetry codes, see: Table 1.

**Fig. 3.**
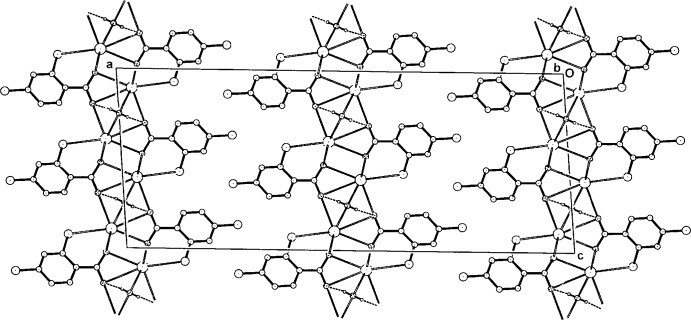
The packing of the structure in the unit cell viewed down [100]. Hydrogen-bonding associations are shown as dashed lines.

## supplementary crystallographic information

### 1. Comment

The structural references for 2,4-dichlorobenzoic acid (2,4-CLBA) or its compounds are absent from the crystallographic literature. The reaction of 2,4-CLBA with potassium carbonate in aqueous ethanol afforded crystals of the title salt, [K_2_(C_7_H_3_Cl_2_O_2_)_2_(H_2_O)]_n_, and the structure is reported herein.

The repeating unit in the polymeric structure consists of two identical irregular KO_6_Cl units related by twofold rotational symmetry, linked by a bridging water molecule lying on the twofold axis. The irregular KO_6_Cl coordination sphere comprises a carboxyl O-atom (O11) and a Cl-atom (Cl2) from a bidentate chelate 2,4-DCBA ligand interaction, four O-atom donors from a doubly bridging bidentate carboxyl *O,O'*-chelate interaction and the bridging water molecule (O1W) (Fig. 1, Table 1). Polymeric extensions in the layered structure, which lies parallel to (100), are through a series of conjoined ring systems including a centrosymmetric carboxyl *O*-bridged cage [K1···K1^ii^ = 4.0310 (9) and a doubly bridged water–carboxyl-*O* cage [K1···K1^v^ = 4.1118 (9) Å] (Figs. 2, 3) [for symmetry code (v): -*x*, *y*, -*z* + 1/2; for symmetry code (ii), see: Table 1].

Coordination complexes involving potassium with aromatic ring-bound Cl donors are uncommon in the crystallographic literature but two polymeric examples have been reported, *viz.* with 4-chlorobenzenesulfonic acid [K—Cl = 3.4051 (14), 3.4969 (14) Å] (Gowda *et al.*, 2007) and with chloranil [K—Cl = 3.4103 (6), 3.5845 (6) Å] (Molčanov *et al.*, 2011). These values are somewhat larger than those in the title complex [3.2670 (7) Å]. Also, a caesium salt having a Cs—Cl bond in a similar bidentate chelate coordination mode with a 2-chloro-substituted aromatic carboxylate ligand is known (Smith, 2013)

The crystal structure of the title complex polymer is stabilized by intra-sheet _water_O—H···O_carboxyl_ hydrogen-bonding interactions (Table 2). A relatively short inversion-related Cl4···Cl4 contact [3.5419 (8) Å] is also present. Although the aromatic ring systems stack down [010] (Fig. 3), no inter-ring π···π interactions are present [minimum ring centroid separation = 4.3407 (3) Å, the *b*-cell parameter].

In the 2,4-DCBA ligand the carboxylate group is significantly rotated out of the plane of the benzene ring [torsion angle C2—C1—C11—O11 = 138.2 (4)°] which is comparable with that in the ammonium salt (also a hemihydrate) [-137.2 (3)°] (Smith, 2014).

### 2. Experimental

The title compound was synthesized by heating together for 10 minutes, 0.5 mmol of 2,4-dichlorobenzoic acid and 0.5 mmol of K_2_CO_3_ in 15 ml of 10% ethanol–water at boiling temperature. Partial room temperature evaporation of the solution gave colourless crystal plates of the title complex from which a specimen was cleaved for the X-ray analysis.

### 3. Refinement

Carbon-bound hydrogen atoms were placed in calculated positions [C—H = 0.95 Å] and allowed to ride in the refinement, with *U*_iso_(H) = 1.2*U*_eq_(C). The hydrogen atom of the coordinating water molecule was located in a difference-Fourier synthesis but was subsequently allowed to ride, with *U*_iso_(H) = 1.5*U*_eq_(O).

### Crystal data

**Table:**

[K_2_(C_7_H_3_Cl_2_O_2_)_2_(H_2_O)]	*F*(000) = 952
*M**_r_* = 476.20	*D*_x_ = 1.814 Mg m^−^^3^
Monoclinic, *C*2/*c*	Mo *K*α radiation, λ = 0.71073 Å
Hall symbol: -C 2yc	Cell parameters from 2539 reflections
*a* = 31.520 (2) Å	θ = 3.6–28.5°
*b* = 4.3407 (3) Å	µ = 1.18 mm^−^^1^
*c* = 12.7849 (9) Å	*T* = 200 K
β = 94.427 (6)°	Plate, colourless
*V* = 1744.0 (2) Å^3^	0.35 × 0.35 × 0.04 mm
*Z* = 4	

### Data collection

**Table:**

Oxford diffraction Gemini-S CCD-detector diffractometer	1714 independent reflections
Radiation source: fine-focus sealed tube	1534 reflections with *I* > 2σ(*I*)
Graphite monochromator	*R*_int_ = 0.084
Detector resolution: 16.077 pixels mm^-1^	θ_max_ = 26.0°, θ_min_ = 3.4°
ω–scans	*h* = −38→38
Absorption correction: multi-scan (*CrysAlis PRO*; Agilent, 2012)	*k* = −5→5
*T*_min_ = 0.706, *T*_max_ = 0.980	*l* = −15→15
9909 measured reflections	

### Refinement

**Table:**

Refinement on *F*^2^	Primary atom site location: structure-invariant direct methods
Least-squares matrix: full	Secondary atom site location: difference Fourier map
*R*[*F*^2^ > 2σ(*F*^2^)] = 0.031	Hydrogen site location: inferred from neighbouring sites
*wR*(*F*^2^) = 0.081	H-atom parameters constrained
*S* = 1.09	*w* = 1/[σ^2^(*F*_o_^2^) + (0.0375*P*)^2^ + 0.3668*P*] where *P* = (*F*_o_^2^ + 2*F*_c_^2^)/3
1714 reflections	(Δ/σ)_max_ < 0.001
114 parameters	Δρ_max_ = 0.37 e Å^−^^3^
0 restraints	Δρ_min_ = −0.23 e Å^−^^3^

### Special details

**Table:**

Geometry. Bond lengths, angles *etc*. have been calculated using the rounded fractional coordinates. All su's are estimated from the variances of the (full) variance-covariance matrix. The cell e.s.d.'s are taken into account in the estimation of distances, angles and torsion angles
Refinement. Refinement of *F*^2^ against ALL reflections. The weighted *R*-factor *wR* and goodness of fit *S* are based on *F*^2^, conventional *R*-factors *R* are based on *F*, with *F* set to zero for negative *F*^2^. The threshold expression of *F*^2^ > σ(*F*^2^) is used only for calculating *R*-factors(gt) *etc*. and is not relevant to the choice of reflections for refinement. *R*-factors based on *F*^2^ are statistically about twice as large as those based on *F*, and *R*- factors based on ALL data will be even larger.

### Fractional atomic coordinates and isotropic or equivalent isotropic displacement parameters (Å^2^)

**Table:**

	*x*	*y*	*z*	*U*_iso_*/*U*_eq_	
K1	0.03304 (1)	0.72596 (10)	0.39507 (3)	0.0247 (2)	
Cl2	0.12895 (2)	0.01261 (12)	0.42912 (4)	0.0310 (2)	
Cl4	0.25235 (1)	0.66649 (13)	0.63591 (4)	0.0323 (2)	
O1W	0.00000	0.3018 (4)	0.25000	0.0308 (7)	
O11	0.05794 (4)	0.1264 (4)	0.69714 (11)	0.0316 (5)	
O12	0.04771 (4)	0.2297 (3)	0.52587 (12)	0.0277 (4)	
C1	0.11633 (6)	0.3174 (4)	0.61306 (15)	0.0207 (6)	
C2	0.14444 (6)	0.2455 (4)	0.53714 (15)	0.0216 (6)	
C3	0.18614 (6)	0.3470 (5)	0.54411 (16)	0.0238 (6)	
C4	0.20012 (6)	0.5325 (5)	0.62714 (16)	0.0240 (6)	
C5	0.17357 (6)	0.6139 (5)	0.70386 (16)	0.0279 (6)	
C6	0.13220 (6)	0.5016 (5)	0.69632 (16)	0.0255 (6)	
C11	0.07044 (6)	0.2136 (4)	0.61080 (16)	0.0217 (6)	
H3	0.20480	0.28990	0.49260	0.0290*	
H5	0.18340	0.74380	0.76040	0.0330*	
H6	0.11410	0.55210	0.74980	0.0310*	
H11W	0.01850	0.19190	0.22930	0.0460*	

### Atomic displacement parameters (Å^2^)

**Table:**

	*U*^11^	*U*^22^	*U*^33^	*U*^12^	*U*^13^	*U*^23^
K1	0.0248 (3)	0.0267 (3)	0.0228 (3)	−0.0016 (2)	0.0024 (2)	−0.0007 (2)
Cl2	0.0301 (3)	0.0387 (3)	0.0251 (3)	−0.0074 (2)	0.0079 (2)	−0.0099 (2)
Cl4	0.0208 (3)	0.0434 (3)	0.0326 (3)	−0.0062 (2)	0.0021 (2)	−0.0011 (2)
O1W	0.0339 (12)	0.0233 (11)	0.0356 (12)	0.0000	0.0051 (9)	0.0000
O11	0.0290 (8)	0.0385 (9)	0.0285 (8)	−0.0052 (7)	0.0107 (6)	0.0029 (7)
O12	0.0215 (7)	0.0325 (8)	0.0289 (8)	−0.0002 (6)	0.0002 (6)	−0.0023 (6)
C1	0.0203 (10)	0.0220 (10)	0.0198 (10)	0.0023 (8)	0.0022 (7)	0.0044 (8)
C2	0.0248 (10)	0.0217 (10)	0.0183 (10)	0.0010 (8)	0.0019 (8)	0.0018 (8)
C3	0.0229 (10)	0.0266 (11)	0.0226 (10)	0.0027 (8)	0.0064 (8)	0.0021 (9)
C4	0.0176 (9)	0.0287 (11)	0.0256 (10)	−0.0003 (8)	0.0019 (8)	0.0042 (9)
C5	0.0261 (11)	0.0316 (11)	0.0257 (11)	−0.0028 (9)	0.0010 (8)	−0.0056 (9)
C6	0.0238 (10)	0.0310 (12)	0.0222 (10)	0.0012 (8)	0.0045 (8)	−0.0032 (9)
C11	0.0211 (10)	0.0180 (9)	0.0264 (11)	0.0034 (8)	0.0042 (8)	−0.0021 (8)

### Geometric parameters (Å, º)

**Table:**

K1—O1W	2.7597 (12)	O1W—H11W^iv^	0.8100
K1—O12	2.7443 (15)	C1—C11	1.513 (3)
K1—Cl2^i^	3.2670 (7)	C1—C2	1.399 (3)
K1—O12^i^	2.7699 (15)	C1—C6	1.393 (3)
K1—O11^ii^	3.0826 (14)	C2—C3	1.383 (3)
K1—O12^ii^	2.8168 (14)	C3—C4	1.377 (3)
K1—O11^iii^	2.7815 (15)	C4—C5	1.384 (3)
Cl2—C2	1.7503 (19)	C5—C6	1.389 (3)
Cl4—C4	1.741 (2)	C3—H3	0.9500
O11—C11	1.259 (2)	C5—H5	0.9500
O12—C11	1.256 (2)	C6—H6	0.9500
O1W—H11W	0.8100		
			
O1W—K1—O12	85.58 (4)	K1^v^—O12—C11	122.38 (11)
Cl2^i^—K1—O1W	129.86 (2)	K1^ii^—O12—C11	99.47 (12)
O1W—K1—O12^i^	165.73 (4)	K1^v^—O12—K1^ii^	99.05 (4)
O1W—K1—O11^ii^	65.80 (4)	K1—O1W—H11W	112.00
O1W—K1—O12^ii^	88.98 (3)	K1—O1W—H11W^iv^	115.00
O1W—K1—O11^iii^	70.16 (4)	K1^iv^—O1W—H11W	115.00
Cl2^i^—K1—O12	96.15 (3)	H11W—O1W—H11W^iv^	108.00
O12—K1—O12^i^	103.85 (4)	K1^iv^—O1W—H11W^iv^	112.00
O11^ii^—K1—O12	120.28 (4)	C2—C1—C6	116.67 (17)
O12—K1—O12^ii^	87.10 (4)	C2—C1—C11	125.17 (17)
O11^iii^—K1—O12	133.56 (5)	C6—C1—C11	118.16 (17)
Cl2^i^—K1—O12^i^	60.61 (3)	C1—C2—C3	122.28 (18)
Cl2^i^—K1—O11^ii^	142.72 (4)	Cl2—C2—C3	116.16 (15)
Cl2^i^—K1—O12^ii^	141.14 (3)	Cl2—C2—C1	121.55 (14)
Cl2^i^—K1—O11^iii^	73.16 (3)	C2—C3—C4	118.81 (18)
O11^ii^—K1—O12^i^	100.01 (4)	Cl4—C4—C3	119.31 (15)
O12^i^—K1—O12^ii^	80.95 (4)	C3—C4—C5	121.38 (18)
O11^iii^—K1—O12^i^	108.77 (5)	Cl4—C4—C5	119.32 (16)
O11^ii^—K1—O12^ii^	44.22 (4)	C4—C5—C6	118.51 (19)
O11^ii^—K1—O11^iii^	85.62 (4)	C1—C6—C5	122.31 (18)
O11^iii^—K1—O12^ii^	129.57 (4)	O11—C11—C1	115.87 (17)
K1^v^—Cl2—C2	121.60 (7)	O12—C11—C1	118.73 (17)
K1—O1W—K1^iv^	96.31 (6)	O11—C11—O12	125.36 (17)
K1^ii^—O11—C11	86.96 (11)	C2—C3—H3	121.00
K1^vi^—O11—C11	149.09 (14)	C4—C3—H3	121.00
K1^ii^—O11—K1^vi^	88.89 (4)	C4—C5—H5	121.00
K1—O12—C11	128.95 (11)	C6—C5—H5	121.00
K1—O12—K1^v^	103.85 (5)	C1—C6—H6	119.00
K1—O12—K1^ii^	92.90 (4)	C5—C6—H6	119.00
			
O12—K1—O1W—K1^iv^	170.99 (3)	K1^v^—Cl2—C2—C3	178.56 (12)
O1W—K1—O12—C11	166.16 (15)	K1^ii^—O11—C11—O12	−20.38 (19)
O1W—K1—O12—K1^v^	10.86 (3)	K1^ii^—O11—C11—C1	157.18 (14)
O1W—K1—O12—K1^ii^	−89.20 (3)	K1^vi^—O11—C11—O12	−103.1 (3)
Cl2^i^—K1—O12—C11	36.50 (15)	K1^vi^—O11—C11—C1	74.5 (3)
Cl2^i^—K1—O12—K1^v^	−118.80 (4)	K1—O12—C11—O11	124.28 (17)
Cl2^i^—K1—O12—K1^ii^	141.14 (3)	K1—O12—C11—C1	−53.2 (2)
O12^i^—K1—O12—C11	−24.70 (16)	K1^v^—O12—C11—O11	−84.4 (2)
O12^i^—K1—O12—K1^v^	−180.00 (4)	K1^v^—O12—C11—C1	98.07 (16)
O12^i^—K1—O12—K1^ii^	79.94 (4)	K1^ii^—O12—C11—O11	22.7 (2)
O11^ii^—K1—O12—C11	−135.26 (15)	K1^ii^—O12—C11—C1	−154.80 (13)
O11^ii^—K1—O12—K1^v^	69.44 (6)	C6—C1—C2—Cl2	179.26 (15)
O11^ii^—K1—O12—K1^ii^	−30.62 (6)	C6—C1—C2—C3	0.9 (3)
O12^ii^—K1—O12—C11	−104.64 (15)	C11—C1—C2—Cl2	−1.4 (3)
O12^ii^—K1—O12—K1^v^	100.06 (5)	C11—C1—C2—C3	−179.75 (18)
O12^ii^—K1—O12—K1^ii^	0.00 (3)	C2—C1—C6—C5	1.0 (3)
O11^iii^—K1—O12—C11	109.06 (16)	C11—C1—C6—C5	−178.47 (18)
O11^iii^—K1—O12—K1^v^	−46.24 (7)	C2—C1—C11—O11	138.2 (2)
O11^iii^—K1—O12—K1^ii^	−146.30 (5)	C2—C1—C11—O12	−44.1 (3)
O12—K1—Cl2^i^—C2^i^	−82.73 (8)	C6—C1—C11—O11	−42.5 (2)
O12—K1—O12^i^—K1^i^	180.00 (5)	C6—C1—C11—O12	135.27 (19)
O12—K1—O12^i^—C11^i^	22.63 (14)	Cl2—C2—C3—C4	179.65 (16)
O12—K1—O11^ii^—K1^iv^	−112.99 (5)	C1—C2—C3—C4	−1.9 (3)
O12—K1—O11^ii^—C11^ii^	36.37 (13)	C2—C3—C4—Cl4	−179.10 (16)
O12—K1—O12^ii^—K1^ii^	0.00 (4)	C2—C3—C4—C5	1.1 (3)
O12—K1—O12^ii^—C11^ii^	−130.28 (11)	Cl4—C4—C5—C6	−179.16 (16)
O12—K1—O11^iii^—K1^iv^	105.31 (5)	C3—C4—C5—C6	0.7 (3)
O12—K1—O11^iii^—C11^iii^	23.1 (3)	C4—C5—C6—C1	−1.7 (3)
K1^v^—Cl2—C2—C1	0.07 (18)		

Symmetry codes: (i) *x*, *y*+1, *z*; (ii) −*x*, −*y*+1, −*z*+1; (iii) *x*, −*y*+1, *z*−1/2; (iv) −*x*, *y*, −*z*+1/2; (v) *x*, *y*−1, *z*; (vi) *x*, −*y*+1, *z*+1/2.

### Hydrogen-bond geometry (Å, º)

**Table:**

*D*—H···*A*	*D*—H	H···*A*	*D*···*A*	*D*—H···*A*
O1*W*—H11*W*···O11^vii^	0.81	1.92	2.7271 (19)	169

Symmetry code: (vii) *x*, −*y*, *z*−1/2.
